# Supplementary material for: Extraction of robust functional connectivity patterns across psychiatric disorders using principal component analysis-based feature selection
Source: Imaging Neurosci (Camb). 2026 Feb 3;4:IMAG.a.1121. doi: 10.1162/IMAG.a.1121 (PMC12869322; doi:10.1162/IMAG.a.1121)
Supplement: Supplementary Text [file IMAG.a.1121_Suppl_Text.pdf]

Supplementary Text S1: Travelling subject harmonization method

We estimated the participant factor (***p***), measurement bias (***m***), sampling biases ($\boldsymbol{s}_{\mathrm{hc}}$, $\boldsymbol{s}_{\mathrm{ssd}}$, $\boldsymbol{s}_{\mathrm{mdd}}$), and psychiatric disorder factor (***d***) by fitting the regression model to the functional connectivity (FC) values of all participants from both the discovery and travelling subject datasets, following the same method as in our previous study (A. Yamashita et al. 2019). As patients with autism spectrum disorder (ASD) were from only one site, we did not need to assume a sampling bias for ASD. For each FC, the regression model was formulated as follows:

$$Connectivity = {x_m}^T m + {x_{s_{hc}}}^T s_{hc} + {x_{s_{scz}}}^T s_{scz} + {x_{s_{mdd}}}^T s_{mdd} + {x_d}^T d + {x_p}^T p + const + \epsilon \text{ (Eq. S1)}$$

$$such\ that \sum_{j}^{9} p_j = 0, \sum_{k}^{4} m_k = 0, \sum_{k}^{4} s_{hc_k} = 0, \sum_{k}^{3} s_{scz_k} = 0, \sum_{k}^{3} s_{mdd_k} = 0, d_1(HC) = 0$$

where m is the measurement bias (4 sites × 1), s_{hc} is the sampling bias for healthy controls (HCs) (4 sites × 1), s_{scz} is the sampling bias for patients with schizophrenia (SCZ), s_{mdd} is the sampling bias for patients with major depressive disorder (MDD), d is the disorder factor (3 disorders × 1), p is the participant factor (9 travelling subjects × 1), $const$ is the average FC value across all participants from all sites, and $\epsilon \sim \mathcal{N}(0, \gamma^{-1})$ denotes noise. A harmonized FC value was obtained by subtracting the estimated measurement bias from the following equation:

$$Connectivity^{Harmonised} = Connectivity - {x_m}^t \grave{m} \qquad \text{(Eq. S2)}$$

where $\grave{m}$ denotes the estimated measurement bias.

Supplementary Text S2: Construction of the network marker and regularization-based feature selection

We constructed a brain network marker for MDD that distinguished between HCs and patients with MDD using the discovery dataset, based on 71,631 FC values, following the same method as in our previous study (A. Yamashita et al. 2020; Okada et al. 2023). To construct the network marker, we applied a machine learning technique called logistic regression with Least Absolute Shrinkage and Selection Operator (LASSO) regularization, as we assumed that psychiatric disorder factors were associated with a specific subset of connections. A logistic function was used to define the probability of a participant belonging to the MDD class as follows:

$$P_{sub}(y_{sub} = 1|\boldsymbol{c}_{sub}; \boldsymbol{w}) = \frac{1}{1+\exp(-\boldsymbol{w}^{\mathrm{T}}\boldsymbol{c}_{sub})}, \text{ (Eq. S3)}$$

in which $\boldsymbol{y}_{\boldsymbol{sub}}$ represents the class label (MDD, y = 1; HC, y = 0) of a participant, $\boldsymbol{c}_{\boldsymbol{sub}}$ represents an FC vector for a given participant, and w represents the weight vector. The weight vector w was determined to minimize

$$J(\mathbf{w}) = -\frac{1}{n_{sub}}\sum_{j=1}^{n_{sub}} \log P_j(y_j = 1|\boldsymbol{c}_j; \boldsymbol{w}) + \lambda \, \|\boldsymbol{w}\|_1, \text{ (Eq. S4)}$$

in which $\|\boldsymbol{w}\|_{\mathbf{1}} = \sum_i^N|\boldsymbol{w}_i|$ and $\boldsymbol{\lambda}$ represent hyperparameters that control the amount of shrinkage applied to the estimates. To determine the weights of the logistic regression model and hyperparameter λ, we employed a nested cross-validation (CV) approach. In this process, the discovery dataset was first split into a training set (9 out of 10 folds) for model training and test set (1 out of 10 folds) for evaluation. To address potential biases caused by the imbalance between the number of patients with MDD and HCs, we applied an undersampling technique. Specifically, we randomly selected approximately 125 MDD patients and 125 HCs from the training set, and the classifier's performance was assessed using the test set. Furthermore, to ensure comparable age distributions between the MDD and HC groups within each subsample, we adjusted the mean age during the undersampling and subsampling steps. Given that only a subset of the training data was used after undersampling, we repeated this random sampling procedure 10 times (subsampling). For each subsample, a model was trained while tuning the regularization parameter within the inner loop of the nested CV, ultimately producing 10 classifiers.

For the inner loop, we used the "*lassoglm*" function in MATLAB (R2016b, Mathworks, USA) with parameters set to "NumLambda = 25" and "CV = 10". The regularization path was defined by first determining the maximum λ ($\lambda_{\max}$), which ensured that the optimal solution was an all-zero vector. A total of 25 λ values were then selected at equal intervals from 0 to $\lambda_{\max}$. The final λ was chosen using the one-standard-error rule, which selects the largest λ within one standard deviation of the minimum prediction error. The classifier output (diagnostic probability) was averaged across the 10 models, and individuals with a diagnostic probability > 0.5 were classified as MDD patients. The area under the curve (AUC) was computed using the "perfcurve" function in MATLAB. In addition, we evaluated classification performance using the accuracy, sensitivity, specificity, positive predictive value (PPV), and negative predictive value (NPV). The Matthews correlation coefficient (MCC) was also calculated to assess model performance in imbalanced datasets. To evaluate the generalizability of the brain network marker, we applied the trained

classifiers to an independent validation dataset. Since 100 classifiers were generated through 10-fold CV × 10 subsamplings, all trained models were used to classify the validation dataset. The final diagnostic probability for each participant was obtained by averaging the outputs of the 100 classifiers, and individuals with a probability > 0.5 were classified as MDD patients.

To identify the most diagnostically relevant FCs, we examined the frequency with which each FC was selected by LASSO during the 10-fold CV process. An FC was considered important if its selection frequency exceeded the chance level, as determined by a permutation test (regularization-based feature selection). Specifically, diagnostic labels in the discovery dataset were permuted, and the entire 10-fold CV and 10-subsampling procedure was repeated 100 times. The selection count for each connection across 10-fold CV × 10 subsamplings (maximum 100 times) was used as a statistic for each permutation dataset. To control for multiple comparisons, we established a null distribution by taking the maximum selection count across all functional connections and set the statistical significance threshold at $P < 0.05$ (one-sided). FCs selected at least 17 times out of 100 were considered diagnostically significant.

Supplementary Figure S1. Schematic representation of the procedure for training the MDD classifier: The MDD

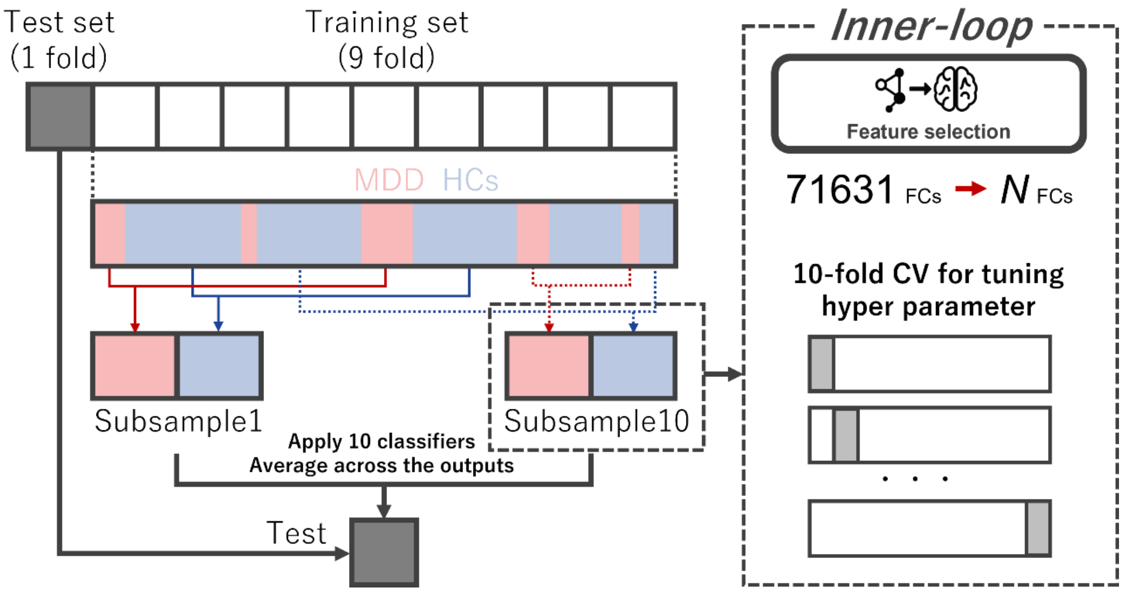

classifier was constructed using a nested cross-validation procedure in the discovery dataset. We also used undersampling and subsampling techniques. Feature selection was performed in the inner loop.

Supplementary Text S3: FC variation analysis in the traveling subject datasets

To assess the impact of experimental factors, such as participants, scanners, and imaging protocols, on FC, we applied a linear fixed-effects model to each FC, following the same method as in our previous study (O. Yamashita et al. 2024). This approach enabled us to estimate the extent to which these factors influenced FC. Specifically, we used a three-factor model—including participant, scanner, and imaging protocol—when analyzing the BMB traveling subject dataset, while for the SRPBS traveling subject dataset, we employed a two-factor model consisting of participant and scanner effects.

z_{nc} represents the z-transformed FC strength for a given run n and a specific FC c, where N is the total number of runs and C is the total number of FCs. We defined $\boldsymbol{z}_c = (z_{1c}, z_{2c}, \dots, z_{nc})$ as a column vector containing the FC strengths across all runs for a particular FC c. In the case of the three-factor model, the relationship was modeled using a linear regression equation with three explanatory variables:

$$\boldsymbol{z}_c = X_p \boldsymbol{\beta}_c^p + X_{prot} \boldsymbol{\beta}_c^{prot} + X_{scan} \boldsymbol{\beta}_c^{scan} + \boldsymbol{\epsilon}_c, \text{(Eq. S5)}$$

Here, the three factors were treated as categorical variables, represented by binary matrices $X_p, X_{prot},$ and X_{scan}. For instance, the participant-factor matrix X_p was structured as an $N \times P$ matrix (where P is the total number of participants), with its (i, j) element set to 1 if participant i was involved in run j, and otherwise it was to 0. The parameter vector $\boldsymbol{\beta}_c^p$, with dimensions $P \times 1$, captured the corresponding effect magnitude for each participant. Similar definitions were applied to the protocol and scanner factors. The term $\boldsymbol{\epsilon}_c$ represented residuals that could not be accounted for by the linear combination of these three factors.

The model (Eq. S5) can be rewritten in a more compact form:

$$\boldsymbol{z}_c = \boldsymbol{X}\boldsymbol{\beta}_c + \boldsymbol{\epsilon}_c, \text{(Eq. S6)}$$

where $\boldsymbol{X} = [X_p \; X_{prot} \; X_{scan}]$ and $\boldsymbol{\beta}_c = \left[\boldsymbol{\beta}_c^{p^t} \; \boldsymbol{\beta}_c^{prot^t} \; \boldsymbol{\beta}_c^{scan^t}\right]^t$ are the combined explanatory matrix and parameter vector, respectively. Using the least squares method, the parameter vector $\boldsymbol{\beta}_c$ was estimated by solving the normal equation:

$$\boldsymbol{X}^t \boldsymbol{X} \boldsymbol{\beta}_c = \boldsymbol{X}^t \boldsymbol{z}_c, \text{(Eq. S7)}$$

Since all three factors were categorical, the matrix $\boldsymbol{X}$ was not of full rank. This could be verified by summing the columns of $X_p, X_{prot},$ and X_{scan}, which would result in a vector of ones. Consequently, the linear equation (Eq. S7) lacked a unique solution (and the inverse of $\boldsymbol{X}^t\boldsymbol{X}$ does not exist). Instead, the least squares solution was obtained using the Moore-Penrose pseudo-inverse:

$$\boldsymbol{\beta}_c = (\boldsymbol{X}^t\boldsymbol{X})^{\dagger}\boldsymbol{X}^t\boldsymbol{z}_c, \text{(Eq. S8)}$$

This solution minimized the L2-norm. Based on statistical analysis, the baseline values for each factor were arbitrary, and only the relative differences within each factor had meaningful interpretability. Therefore, we computed the FC variations attributed to the participant (or individual subject), protocol, and scanner factors by determining the standard deviation of $\boldsymbol{\beta}_c^p$, $\boldsymbol{\beta}_c^{prot}$, and $\boldsymbol{\beta}_c^{scan}$ across the members within each factor, respectively. The pair-wise distance matrix between members of the scanner type and imaging protocol factors was computed by calculating the mean absolute difference between corresponding estimated parameters across all FCs.

Supplementary Text S4: Robustness analyses

Application of PCA weights to the validation dataset

In the main analysis, PCA was run separately in the discovery and validation datasets to test the robustness of the feature extraction process. To further test the robustness of the extracted components themselves, we additionally applied the PCA weights obtained from the discovery dataset directly to the validation dataset. The resulting PCA scores still showed a significant difference between the MDD and HC groups in the validation dataset (two-sample *t*-test, discovery dataset: $t = -6.58$, $p < 0.001$, Cohen's $d = -0.63$; validation dataset: $t = -8.27$, $p < 0.001$, Cohen's $d = -0.76$). This result indicates that the extracted components generalize across datasets, strengthening the evidence for their robustness.

Supplementary Figure S2. Reproducibility test by applying PC weights derived from the discovery dataset to the

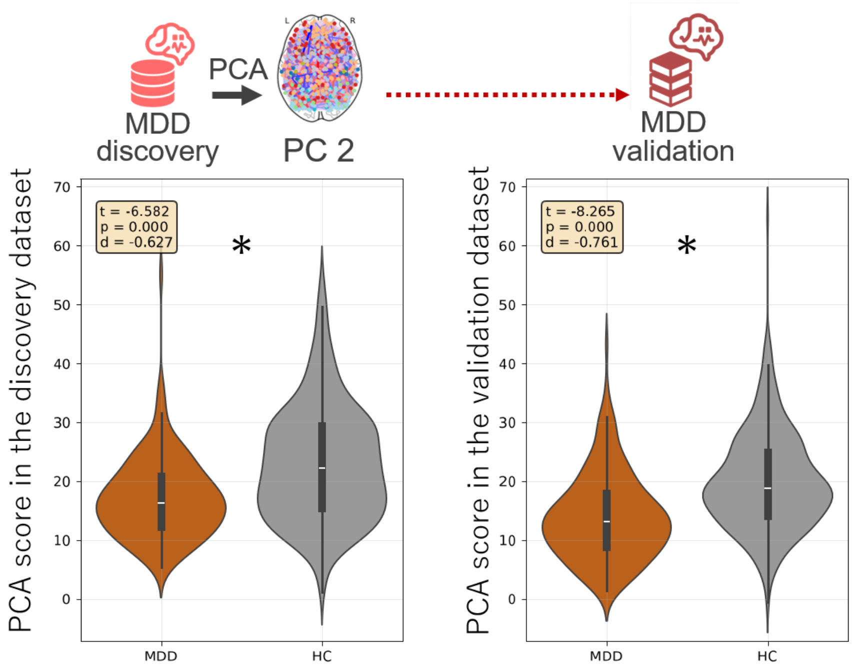

validation dataset in MDD: The left panel shows violin plots of PCA scores for MDD and HC groups in the discovery dataset, while the right panel shows violin plots of PCA scores for MDD and HC groups in the validation dataset.

Alternative brain parcellation (Schaefer400 atlas).

To evaluate whether the findings depend on the choice of brain atlas, we repeated the analyses using the Schaefer 400 parcellation (Schaefer et al. 2018) instead of the Glasser atlas. The results were consistent: the second principal component again showed a significant difference in PCA scores between patients with MDD and healthy controls, and the functional connections selected were qualitatively similar in both the discovery and validation datasets. This supports the robustness of our method across different brain templates.

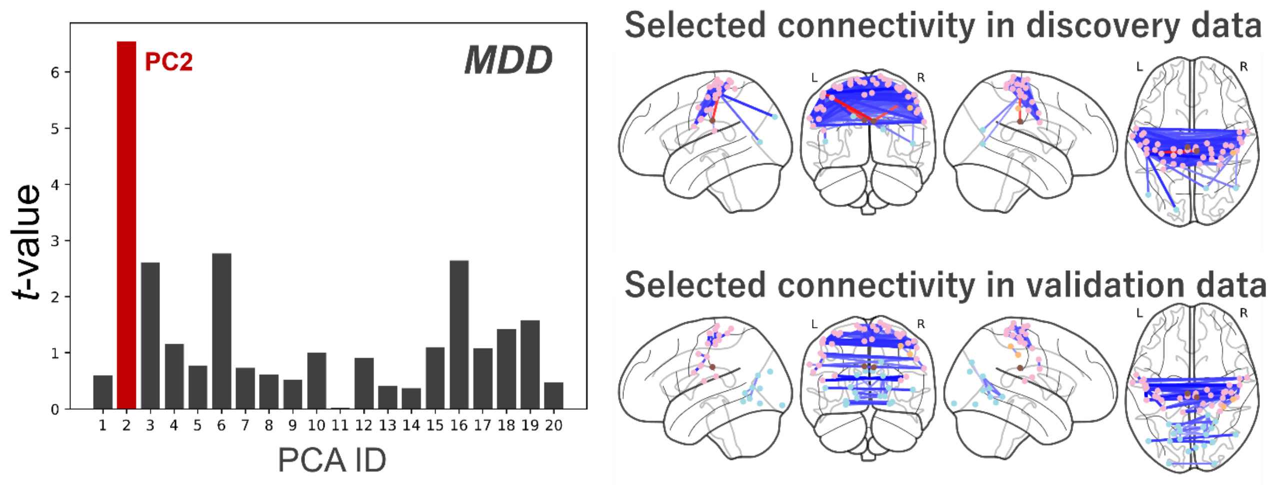

Supplementary Figure S3. Results using the Schaefer400 atlas in MDD: The left panel shows the *t*-values for group differences between patients with MDD and healthy controls across PCs. The upper-right panel illustrates the FCs selected in the discovery dataset, and the lower-right panel illustrates the FCs selected in the validation dataset.

Leave-one-site-out analysis.

To examine whether site differences influenced the results, we repeated the PCA after sequentially excluding each of the four sites in the discovery dataset. In all four analyses, PC2 was consistently identified as the MDD-related component. Furthermore, the PCA weights of this component were highly similar to those from the original analysis, with a mean Pearson's correlation of 0.98 (SD 0.0026) across the four analyses. These findings indicate that the diagnosis-related component was robust to site composition and not driven by data from any single site.

Bootstrap analysis

To further evaluate the stability of the components, we performed bootstrapping on the discovery dataset by resampling participants with replacement 100 times. In all bootstrap samples, PC2 was consistently identified as the MDD-related component. Moreover, the PCA weights of this component showed high similarity to those from the original analysis, with a mean Pearson's correlation of 0.95 (min = 0.88, max = 0.97) (Supplementary Fig. S4).

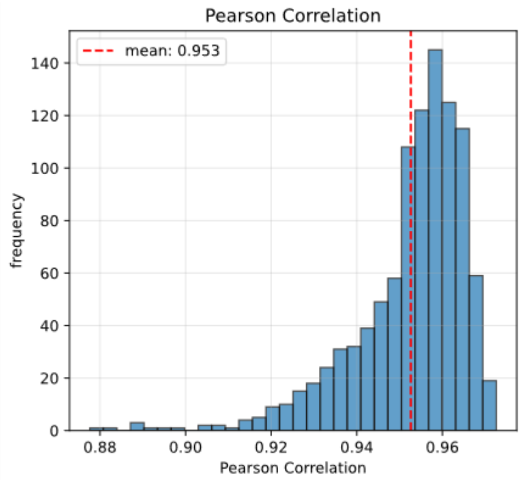

Supplementary Figure S4. Histogram of correlations between bootstrap PC weights and original PC weights (100 resamples) in MDD

Matched-group size procedure

To examine whether the imbalance between patients and healthy controls (HCs) in the discovery dataset influenced the PCA decomposition, we performed an additional matched-group PCA analysis. Specifically, for each disorder, we randomly sampled the same number of HCs as patients (MDD: 138, ASD: 121, SCZ: 102), resulting in datasets of 276, 242, and 204 participants, respectively. For each matched dataset, PCA was performed independently. To examine the stability of the diagnosis-related component, we repeated this procedure 1,000 times using bootstrap resampling of HCs. In each bootstrap iteration, we computed the absolute correlations between the disorder-specific original PC2 weights (i.e., the PC2 weights obtained from the full, imbalanced dataset for that disorder) and each of the first five principal components (PC1–PC5) of the matched PCA. We then identified the component with the highest absolute correlation in each iteration and examined (i) the distribution of these maximum correlations and (ii) the frequency with which each PC (PC1–PC5) was selected as the best-matching component.

Across 1,000 iterations, the MDD, ASD, and SCZ analyses all showed consistently high correlations with the original PC2 weights (Pearson's *r*, mean ± standard deviation: MDD = 0.867 ± 0.048, ASD = 0.848 ± 0.064, SCZ = 0.798 ± 0.076), demonstrating that the diagnosis-related component was robust to group-size imbalance. In addition, regarding which PC showed the highest correlation with the disorder-specific original PC2 weights, PC2 was selected in 994 out of 1,000 iterations for MDD and in 926 iterations for ASD. For SCZ, PC2 and PC3 were selected in 436 and 521 iterations, respectively. Thus, while the diagnosis-related pattern consistently emerged as PC2 in MDD and ASD, it appeared as either PC2 or PC3 in SCZ. This additional analysis further confirms that the diagnosis-related component is not an artifact of group-size imbalance.

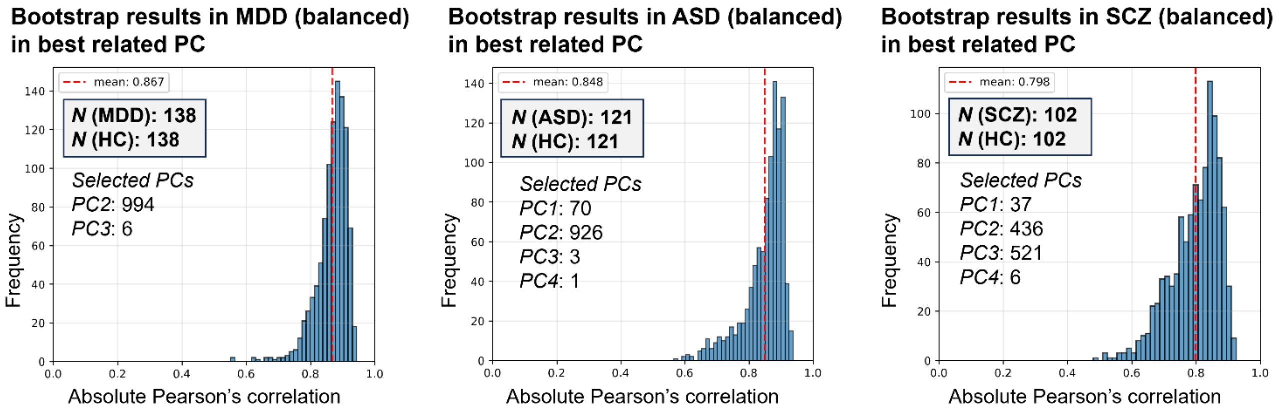

Supplementary Figure S5. Distribution of correlations between the original PC2 weights and the best related PC weights derived from matched-group PCA across 1,000 bootstrap iterations for MDD, ASD, and SCZ.

Stricter scrubbing analysis

We also tested whether motion-related confounds could explain the findings by reanalyzing the data with a more stringent scrubbing criterion (removing time points with FD > 0.2 mm). Participants with less than 4 minutes of data remaining after scrubbing were excluded. As a result, the discovery dataset was reduced from 683 participants (HC = 545, MDD = 138) to 499 (HC = 401, MDD = 98), and the validation dataset from 519 (HC = 338, MDD = 181) to 388 (HC = 245, MDD = 143). Importantly, even under this stricter criterion, the diagnosis-related PC remained PC2, and the PCA weights showed a very high correlation with those from the original analysis (*r* = 0.97) (Supplementary Figure S5). Taken together, these findings argue against the influence of motion-related confounds and further support the interpretation that the observed PCs reflect shared biological signals and underlying variance structures across cohorts.

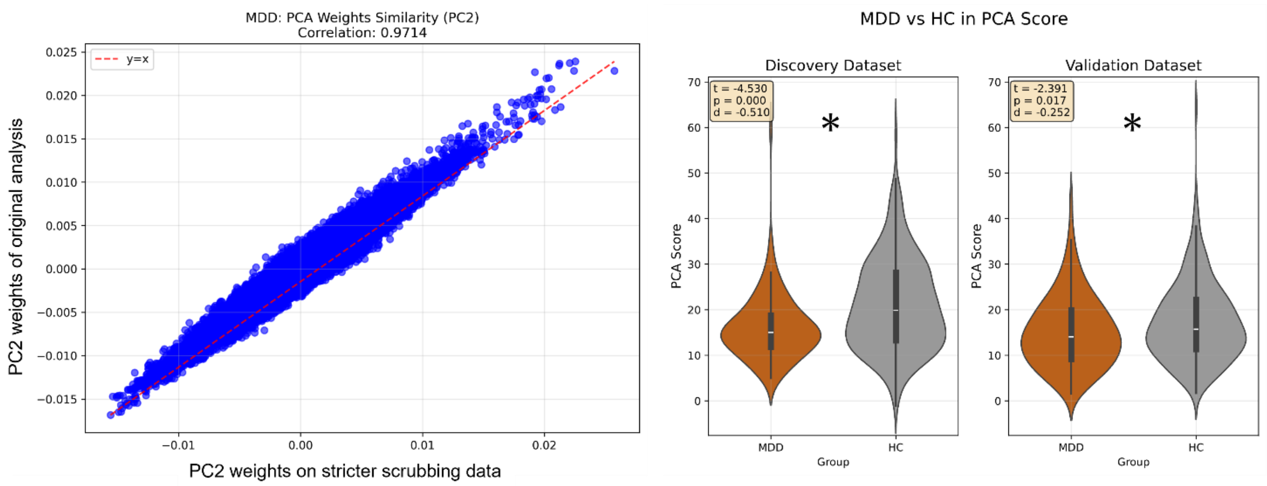

Supplementary Figure S6. Stability of the MDD-related PC under stricter motion scrubbing (FD > 0.2 mm): (Left) Scatter plot showing the similarity of PCA weights for the MDD-related PC (PC2) between the original analysis and the analysis using stricter scrubbing (FD > 0.2 mm). The weights showed a very high correlation ($r = 0.97$). (Right) Violin plots of PCA scores for MDD and HC groups in the discovery dataset and validation dataset under the stricter scrubbing criterion. Significant group differences were consistently observed in both datasets, confirming the robustness of the MDD-related PC.

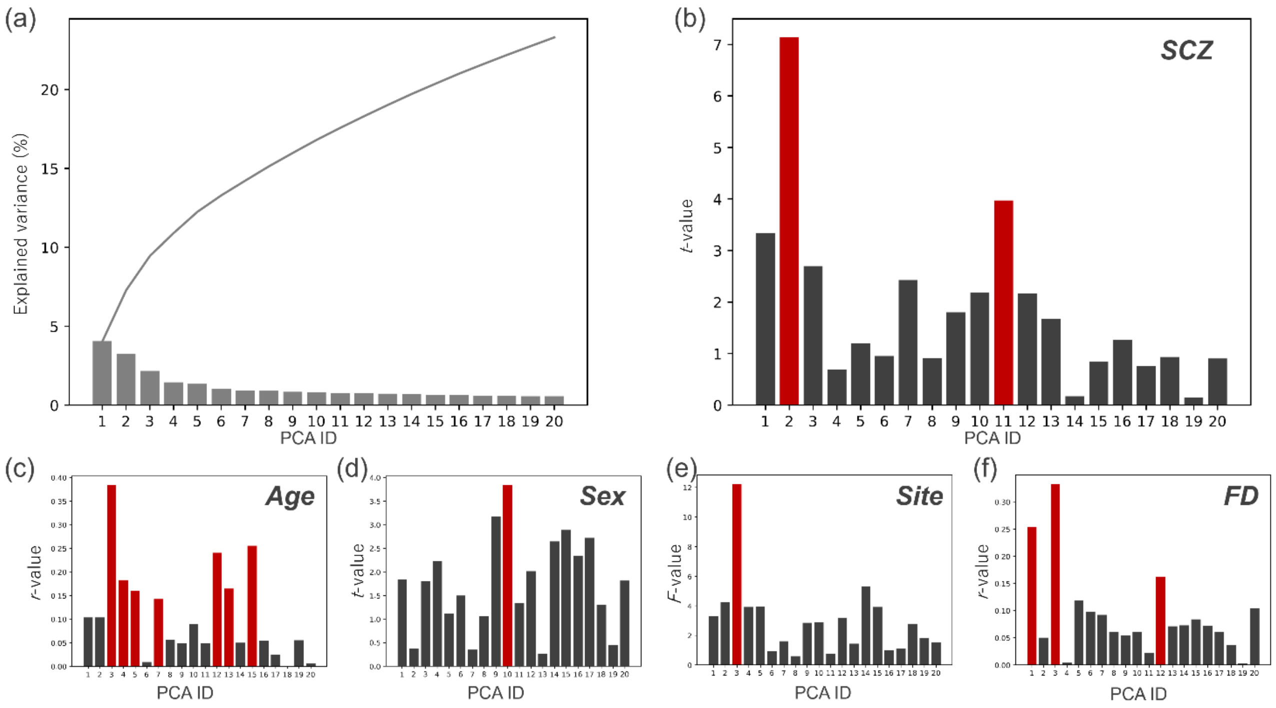

Supplementary Figure S7. Relationship between the principal component (PC) score and factors in the top 20 PCs in the schizophrenia (SCZ) discovery dataset: (a) Explained variance of the top 20 PCs and their cumulative summation. (b–f) Relationship between each factor and the top 20 PCs. (b) Difference in PC scores between HCs and patients with SCZ (*t*-value). (c) Pearson's correlation coefficients between the PC score and age. (d) Difference in PC scores between men and women (*t*-value). (e) Difference in PC scores across imaging sites (*F*-value). (f) Pearson's correlation coefficients between the PC score and head motion (framewise displacement value). The red bar shows a significant relationship with the factor. Here, we only visualized the top 20 PCs for visualization purpose. Of note, we used all PCs for the analyses. PCA: principal component analysis

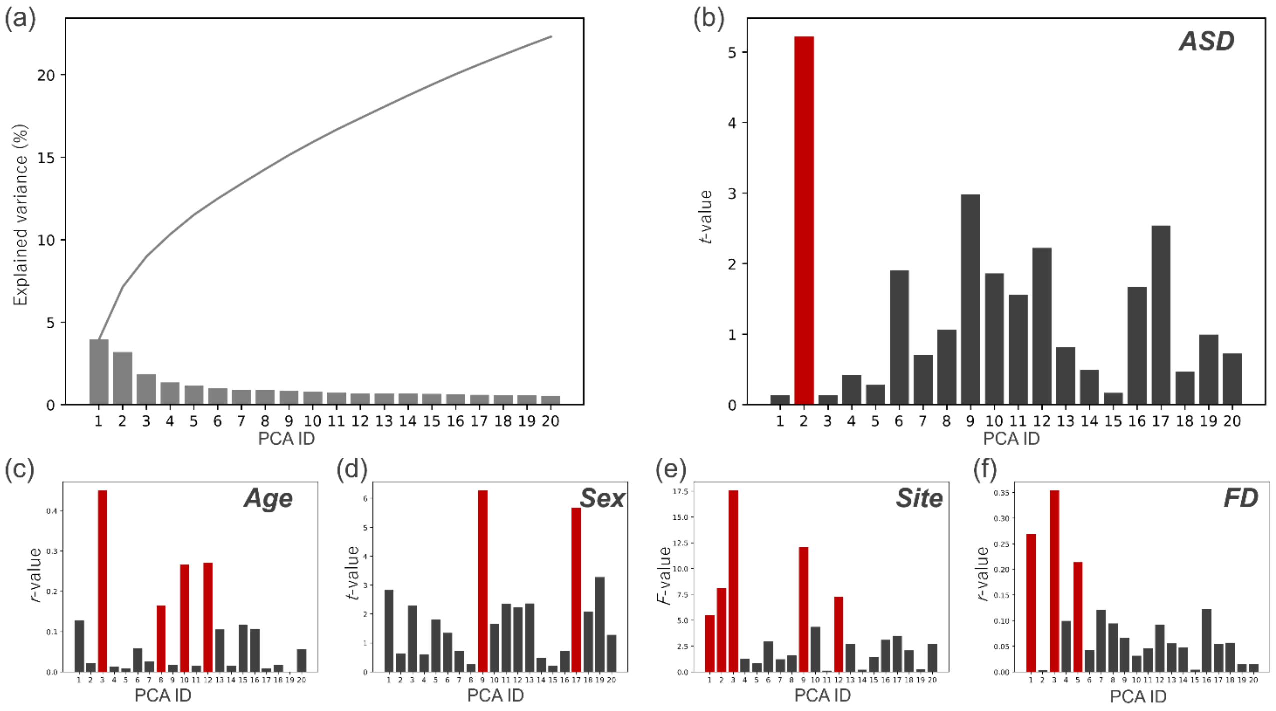

Supplementary Figure S8. Relationship between the principal component (PC) score and factors in the top 20 PCs in the autism (ASD) discovery dataset: (a) Explained variance of the top 20 PCs and their cumulative summation. (b–f) Relationship between each factor and the top 20 PCs. (b) Difference in PC scores between HCs and patients with ASD (*t*-value). (c) Pearson's correlation coefficients between the PC score and age. (d) Difference in PC scores between men and women (*t*-value). (e) Difference in PC scores across imaging sites (*F*-value). (f) Pearson's correlation coefficients between the PC score and head motion (framewise displacement value). The red bar shows a significant relationship with the factor. Here, we only visualized the top 20 PCs for visualization purpose. Of note, we used all PCs for the analyses. PCA: principal component analysis

References

Okada, Go, Toshinori Yoshioka, Ayumu Yamashita, Eri Itai, Satoshi Yokoyama, Toshiharu Kamishikiryo, Hotaka Shinzato, et al. 2023. "Verification of the Brain Network Marker of Major Depressive Disorder: Test-Retest Reliability and Anterograde Generalization Performance for Newly Acquired Data." *Journal of Affective Disorders* 326 (April): 262–66.

Schaefer, Alexander, Ru Kong, Evan M. Gordon, Timothy O. Laumann, Xi-Nian Zuo, Avram J. Holmes, Simon B. Eickhoff, and B. T. Thomas Yeo. 2018. "Local-Global Parcellation of the Human Cerebral Cortex from Intrinsic Functional Connectivity MRI." *Cerebral Cortex (New York, N.Y.: 1991)* 28 (9): 3095–3114.

Yamashita, Ayumu, Yuki Sakai, Takashi Yamada, Noriaki Yahata, Akira Kunimatsu, Naohiro Okada, Takashi Itahashi, et al. 2020. "Generalizable Brain Network Markers of Major Depressive Disorder across Multiple Imaging Sites." *PLoS Biology* 18 (12): e3000966.

Yamashita, Ayumu, Noriaki Yahata, Takashi Itahashi, Giuseppe Lisi, Takashi Yamada, Naho Ichikawa, Masahiro Takamura, et al. 2019. "Harmonization of Resting-State Functional MRI Data across Multiple Imaging Sites via the Separation of Site Differences into Sampling Bias and Measurement Bias." *PLoS Biology* 17 (4): e3000042.

Yamashita, Okito, Ayumu Yamashita, Yuji Takahara, Yuki Sakai, Yasumasa Okamoto, Go Okada, Masahiro Takamura, et al. 2024. "Computational Mechanisms of Neuroimaging Biomarkers Uncovered by Multicenter Resting-State FMRI Connectivity Variation Profile." *BioRxiv*. https://doi.org/10.1101/2024.04.01.587535.

Supplementary Table S1. Participant demographics in the "discovery dataset"

Site	HC			MDD			ASD			SCZ			ALL		
	N	M/F	Age (mean±SD)	N	M/F	Age (mean±SD)	N	M/F	Age (mean±SD)	N	M/F	Age (mean±SD)	N	M/F	Age (mean±SD)
SWA	99	84/15	28.4±7.9	–	–	–	111	96/15	32.0±7.5	18	14/4	42.8±8.6	228	194/34	31.3±8.6
COI	112	44/68	50.9±13.4	62	29/33	44.6±12.4	–	–	–	–	–	–	174	73/101	48.7±13.4
KUT	166	99/67	35.6±13.5	17	11/6	43.9±13.3	–	–	–	48	24/24	41.5±10.4	231	134/97	37.5±13.2
UTO	168	77/91	35.6±17.5	59	35/24	38.2±11.4	10	9/1	37.0±9.6	36	24/12	31.4±10.3	273	145/128	35.7±15.4
Summary	545	304/241	37.4±15.9	138	75/63	41.8±12.4	121	105/16	32.4±7.8	102	62/40	38.2±11.2	906	546/360	37.5±14.3

Abbreviations: HC: healthy control, MDD: major depressive disorder, ASD: autism spectrum disorder, SCZ: schizophrenia, M: Male, F: Female, SD: standard deviation. SWA: Showa University, COI: Centre of Innovation, Hiroshima University, KUT: Kyoto University (TimTrio), UTO: the University of Tokyo

Supplementary Table S2. Participant demographics in the "validation dataset"

Site	HC			MDD			ASD			SCZ			ALL		
	N	M/F	Age (mean±SD)	N	M/F	Age (mean±SD)	N	M/F	Age (mean±SD)	N	M/F	Age (mean±SD)	N	M/F	Age (mean±SD)
HUH	66	29/37	34.6±13.0	57	32/25	43.3±12.2	–	–	–	–	–	–	123	61/62	38.6±13.3
HRC	49	13/36	41.7±11.7	16	6/10	40.5±11.5	–	–	–	–	–	–	65	19/46	41.4±11.5
HKH	28	11/17	45.9±9.3	32	20/12	45.2±11.5	–	–	–	–	–	–	60	31/29	45.5±10.5
KTT	75	48/27	28.9±9.1	–	–	–	–	–	–	52	27/25	37.2±9.4	127	75/52	32.3±10.0
UYA	120	50/70	45.9±19.5	76	35/41	50.2±13.8	–	–	–	–	–	–	196	85/111	47.6±17.6
Summary	338	151/187	39.3±16.1	181	93/88	46.3±13.1	–	–	–	52	27/25	37.2±9.4	571	271/300	41.3±15.1

Abbreviations: HC: healthy control, SCZ: schizophrenia, MDD: major depressive disorder, ASD: autism spectrum disorder, M: Male, F: Female, SD: standard deviation. HUH: Hiroshima University Hospital, HRC: Hiroshima Rehabilitation Center, HKH: Hiroshima Kajikawa Hospital, KTT: Kyoto University (Trio), UYA: Yamaguchi University

Supplementary Table S3. Participant demographics in the "COBRE dataset"

Site	HC			MDD			ASD			SCZ			ALL		
	N	M/F	Age (mean±SD)	N	M/F	Age (mean±SD)	N	M/F	Age (mean±SD)	N	M/F	Age (mean±SD)	N	M/F	Age (mean±SD)
COBRE	45	31/14	32.0±9.6	–	–	–	–	–	–	30	26/4	31.8±12.2	75	57/18	31.9±10.6

Abbreviations: HC: healthy control, MDD: major depressive disorder, ASD: autism spectrum disorder, SCZ: schizophrenia, M: Male, F: Female, SD: standard deviation. COBRE: Centre of Biomedical Research Excellence

Supplementary Table S4. Participant demographics in the "ABIDE-I and -II dataset"

	HC			MDD			ASD			SCZ			ALL		
	N	M/F	Age (mean±SD)	N	M/F	Age (mean±SD)	N	M/F	Age (mean±SD)	N	M/F	Age (mean±SD)	N	M/F	Age (mean±SD)
CMU_a	8	7/1	26.4±4.3	–	–	–	6	6/0	26.2±4.7	–	–	–	14	13/1	26.3±4.3
CMU_b	5	3/2	27.6±8.1	–	–	–	7	5/2	25.9±7.2	–	–	–	12	8/4	26.6±7.3
KKI	32	23/9	10.2±1.3	–	–	–	14	11/3	9.8±1.4	–	–	–	46	34/12	10.0±1.3
Leuven_1	15	15/0	23.3±2.9	–	–	–	14	14/0	21.9±4.1	–	–	–	29	29/0	22.6±3.6
Leuven_2	20	15/5	14.3±1.5	–	–	–	14	11/3	13.7±1.1	–	–	–	34	26/8	14.1±1.4
MaxMun	12	8/4	33.2±9.0	–	–	–	12	9/3	35.5±11.4	–	–	–	24	17/7	34.4±10.1
MaxMun2	18	18/0	23.0±7.5	–	–	–	9	9/0	19.2±12.8	–	–	–	27	27/0	21.7±9.5
NYU	104	78/26	15.8±6.3	–	–	–	76	66/10	14.8±7.0	–	–	–	180	144/36	15.4±6.6
Olin	12	10/2	17.8±3.2	–	–	–	13	11/2	17.2±3.7	–	–	–	25	21/4	17.5±3.4

Pitt	22	19/3	19.1±6.4	–	–	–	19	16/3	19.3±7.2	–	–	–	41	35/6	19.2±6.7
SBL	15	15/0	33.7±6.6	–	–	–	14	14/0	35.3±10.8	–	–	–	29	29/0	34.5±8.7
Trinity	23	23/0	17.5±3.7	–	–	–	21	21/0	17.5±3.2	–	–	–	44	44/0	17.5±3.4
UCLA_1	28	24/4	13.6±2.0	–	–	–	29	25/4	13.6±2.7	–	–	–	57	49/8	13.6±2.3
UCLA_2	12	10/2	12.4±1.0	–	–	–	8	8/0	12.4±2.1	–	–	–	20	18/2	12.4±1.5
UM	75	57/18	14.8±3.6	–	–	–	54	45/9	13.7±2.3	–	–	–	129	102/27	14.3±3.2
USM	41	41/0	21.7±7.5	–	–	–	51	51/0	23.1±7.9	–	–	–	92	92/0	22.5±7.7
Yale	26	19/7	12.8±2.8	–	–	–	26	19/7	12.8±3.0	–	–	–	52	38/14	12.8±2.9
ABIDEII BNI_1	23	23/0	40.0±15.7	–	–	–	24	24/0	37.1±15.8	–	–	–	47	47/0	38.5±15.6
ABIDEII EMC_1	15	12/3	8.1±1.0	–	–	–	14	12/2	8.6±1.3	–	–	–	29	24/5	8.3±1.1
ABIDEII GU_1	30	16/14	10.8±1.8	–	–	–	20	18/2	11.4±1.6	–	–	–	50	34/16	11.0±1.8
ABIDEII IU_1	–	–	–	–	–	–	2	1/1	31.5±14.8	–	–	–	2	1/1	31.5±14.8
ABIDEII KKI_8	186	108/78	10.4±1.2	–	–	–	56	44/12	10.6±1.5	–	–	–	242	152/90	10.4±1.3
ABIDEII KKI_32	78	56/22	10.5±1.2	–	–	–	20	16/4	10.8±1.4	–	–	–	98	72/26	10.5±1.3
ABIDEII KUL_3	–	–	–	–	–	–	2	2/0	20.5±0.7	–	–	–	2	2/0	20.5±0.7
ABIDEII NYU_1	28	26/2	9.5±3.4	–	–	–	43	39/4	10.2±6.0	–	–	–	71	65/6	9.9±5.1
ABIDEII OHSU_1	50	26/24	10.5±1.7	–	–	–	31	25/6	11.6±2.3	–	–	–	81	51/30	10.9±2.0
ABIDEII ONRC_2	32	19/13	24.4±3.5	–	–	–	20	17/3	21.8±3.5	–	–	–	52	36/16	23.4±3.7
ABIDEII TCD_1	18	18/0	16.3±2.8	–	–	–	14	14/0	15.3±3.5	–	–	–	32	32/0	15.9±3.1

ABIDEII UCD_1	13	9/4	15.0±1.6	–	–	–	15	12/3	15.0±2.0	–	–	–	28	21/7	15.0±1.8
ABIDEII UCLA_1	13	9/4	10.0±2.3	–	–	–	11	11/0	12.2±1.7	–	–	–	24	20/4	11.0±2.3
ABIDEII USM_1	15	12/3	24.0±8.1	–	–	–	15	13/2	19.2±6.9	–	–	–	30	25/5	21.6±7.8
Summary	969	719/250	15.3±8.1	–	–	–	674	589/85	16.5±9.1	–	–	–	1643	1308/335	15.8±8.6

Abbreviations: ABIDE: Autism Brain Imaging Data Exchange, HC: healthy control, MDD: major depressive disorder, ASD: autism spectrum disorder, SCZ: schizophrenia, M: Male, F: Female, SD: standard deviation. CMU: Carnegie Mellon University, KKI: Kennedy Krieger Institute, MaxMun: Ludwig Maximilians University Munich, NYU: New York University Langone Medical Center, SBL: Social Brain Lab BCN NeuroImaging Center, University Medical Center Groningen and Netherlands Institute for Neurosciences, UCLA: the University of California, Los Angeles, University of Michigan, USM: University of Utah School of Medicine, BNI: Barrow Neurological Institute, EMC: Erasmus University Medical Center Rotterdam, GU: Georgetown University, IU: Indiana University, KUL: Katholieke Universiteit Leuven, OHSU: Oregon Health and Science University, ONRC: Olin Neuropsychiatry Research Center, Institute of Living at Hartford Hospital, TCD: Trinity Centre for Health Sciences, UCD: University of California Davis,

Supplementary Table S5. Imaging parameters of each imaging site in the discovery, validation and COBRE datasets.

	Discovery dataset				Validation dataset					COBRE
Site	KUT	SWA	COI	UTO	KTT	KUP	HKH	HRC	HUH	COBRE
MRI scanner	Siemens TimTrio	Siemens Verio	Siemens Verio	GE MR750w	Siemens Trio	Siemens Prisma	Siemens Spectra	GE Signa HDxt	GE Signa HDxt	Siemens TimTrio
Magnetic field strength	3.0 T									
Channels per coil	32	12		24	8	64	12	8		
Field of view (mm)	212 × 212				256 × 192	200 × 200	192 × 192	256 × 256		240 × 240
Matrix	64 × 64				64 × 48	100 × 100	64 × 64			
Number of slices	40				30	72	38	32		33
Number of volumes	240				180	320	107	143		150
In-plane resolution (mm)	3.3125 × 3.3125				4 × 4	2 × 2	3 × 3	4 × 4		3.75 × 3.75
Slice thickness (mm)	3.2				4	2	3	4		3.5
Slice gap (mm)	0.8				0					1.05
TR (s)	2.5				2	0.75	2.7	2		
TE (ms)	30				30	36.2	31	27		29
Total scan time	10'00"				6'00"	4'10"	5'00"	4'46"	5'00"	6'00"
Flip angle (degree)	80				90	55	90			75
Slice acquisition order	Ascending				Ascending (interleaved)		Ascending	Ascending (interleaved)		
Phase encoding	P→A		A→P	P→A	A→P	P→A	A→P		P→A	A→P
Eye condition	Fixated				Fixated					Not specified

Supplementary Table S6. Imaging parameters of each imaging site in the ABIDE-I dataset.

Site	CMU	KKI	Leuven	MaxMun	NYU	Olin	Pitt	SBL	Trinity	UCLA	UM	USM	Yale
MRI scanner	Siemens Verio	Philips Achieva	Philips Intera	Siemens Verio	Siemens Allegra	Siemens Allegra	Siemens Allegra	Philips Intera	Philips Achieva	Siemens TrioTim	GE Signa	Siemens TrioTim	Siemens TimTrio
Magnetic field strength	3T	3T	3T	3T	3T	3T	3T	3T	3T	3T	3T	3T	3T
Channels per coil	NA	8	8	NA	NA	NA	NA	NA	8	NA	NA	NA	NA
Field of view (mm)	192x192	256x256	230x230	192x192	240x192	220x220	200x200	220x220	240x240	192x192	220x220	220x220	220x220
Matrix	64x64	84x81	64x64	64x64	80x64	64x64	64x64	80x80	80x80	64x64	64x64	64x64	64x64
Number of slices	21 or 28	47	32	28	33	29	29	38	38	34	40	40	34
Number of volumes	240	156	250	120	180	210	200	200	150	120	300	240	200
In-plane resolution (mm)	3x3	3.05x3.15	3.59x3.59	3x3	3x3	3.4x3.4	3.1x3.1	2.75x2.75	3x3	3x3	3.438x3.438	3.4x3.4	3.4x3.4
Slice thickness (mm)	3	3	4	4	4	4	4	2.72	3.5	4	3	3	4
Slice gap (mm)	1.5	0	0	0.4	0	1	0	0.272	0.35	0	0	0.3	0
TR (s)	1.5 or 2	2.5	1.667	3	2	1.5	1.5	2.2	2	3	2	2	2
TE (ms)	0.03	0.03	0.033	0.03	0.015	0.027	0.025	0.03	0.028	0.028	0.03	0.028	0.025
Total scan time	8:06	6:40	7:06	6:06	6:00	5:15	5:06	7:28	5:06	6:06	10:00	8:06	6:40
Flip angle (degree)	73	75	90	80	90	60	70	80	90	90	90	90	60

Slice acquisition order	Ascending (interleaved)	Ascending (sequential)	Ascending (sequential)	Ascending (interleaved)	Ascending (interleaved)	Ascending (interleaved)	Ascending (interleaved)	Descending (sequential)	Ascending (sequential)	Ascending (interleaved)	Ascending (sequential)	Ascending (interleaved)	Ascending (interleaved)
Phase encoding	AP	AP	AP	AP	RL	AP	AP	AP	AP	AP	AP	AP	AP
Eye condition	Close	Fixated	Close	Close or Open	Fixated	Fixated	Close	Close	Close	Open	Fixated	Open	Open

Supplementary Table S7. Imaging parameters of each imaging site in the ABIDE-II dataset.

Site	BNI	EMC	GU	IU	KKI	KUL	NYU	OHSU	ONRC	TCD	UCD	UCLA	USM
MRI scanner	Philips Ingenia	GE MR750	Siemens TrioTim	Siemens TrioTim	Philips Achieva	Philips Achieva	Siemens Allegra	Siemens TrioTim	Siemens Skyra	Philips Intera Achieva	Siemens TrioTim	Siemens TrioTim	Siemens TrioTim
Magnetic field strength	3T	3T	3T	3T	3T	3T	3T	3T	3T	3T	3T	3T	3T
Channels per coil	15	8	12	32	8 or 32	32	8	12	NA	NA	32	NA	12
Field of view (mm)	240X240	230x230	192x192	220x220	256x256	200x200	240x240	240x240	240x240	240x240	224x224	192x192	220x220
Matrix	64X64	64x64	64x64	64x64	84x81	80x78	80x80	64x64	80x80	80x80	64x64	64x64	64x64
Number of slices	50	37	43	42	47	45	34	36	48	37	36	34	40
Number of volumes	120	160	154	433	128,139,156	162	180	120	947	210	460	120	240
In-plane resolution (mm)	3.75X3.75	3.5938x3.5938	3x3	3.4x3.4	3x3	2.5x2.56	3x3x4	3.8x3.8	3x3	3x3	3.5x3.5	3x3	3.4x3.4
Slice thickness (mm)	4	4	2.5	3.4	3	2.7	3	3.8	3	3.2	4	4	3
Slice gap (mm)	0	0	0.5	0	0	0.4	0	0	0	0.35	0	0	0.3
TR (s)	3	2	2	0.813	2.5	2.5	2	2.5	0.475	2	2	3	2
TE (ms)	0.025	0.03	0.03	0.028	0.03	0.03	0.03	0.03	0.03	0.027	0.024	0.028	0.028
Total scan time	6:09	5:02	5:14	16:21	5:20, 5:47, 6:30	7:00	6:00	5:07	7:37	7:06	15:24	6:06	8:06
Flip angle (degree)	80	85	90	60	75	90	82	90	60	90	90	90	90
Slice acquisition order	Ascending (sequential)	Descending (interleaved)	Ascending (interleave d)	Ascending (interleaved)	Ascending (sequential)	Ascending (sequential)	Ascending (interleaved)	Ascending (interleaved)	Ascending (interleaved)	Descending (sequential)	Ascending (interleaved)	Ascending (interleaved)	Ascending (interleaved)
Phase encoding	AP	AP	AP	AP	AP	AP	RL	AP	RL	AP	AP	AP	AP
Eye condition	Close	Close	Open	Open	Fixated	Fixated	Open	Open	Fixated	Fixated	Open	Open	Open
